# Drug policies and annual opioid overdose mortality in U.S. states with the five worst and five best trends between 2018 and 2023: an ecological study

**DOI:** 10.1016/j.abrep.2026.100760

**Published:** 2026-10-22

**Authors:** Yun-Ting Wang, Said Iftekhar Sadaat, Daniel Ciccarone, Mohammad Karamouzian, Ali Mirzazadeh

**Affiliations:** a Institute for Global Health Sciences, University of California, San Francisco, CA, USA; b Family and Community Medicine, University of California, San Francisco, CA, USA; c Centre on Drug Policy Evaluation, MAP Centre for Urban Health Solutions, St. Michael's Hospital, Toronto, ON, Canada; d Dalla Lana School of Public Health, University of Toronto, Toronto, ON, Canada; e Department of Epidemiology and Biostatistics, University of California, San Francisco, CA, USA

**Keywords:** Drug policy, Opioid overdose, United States

## Abstract

**Purpose:**

To examine state-level opioid overdose mortality trends in the United States and assess their association with drug policy environments.

**Methods:**

This ecological study analyzed opioid-related overdose death data from 2018 to 2023 for all U.S. states using CDC WONDER. Linear regression estimated state-level mortality trends. The five states with the steepest increases and five with the most stable or improving trends were identified. Drug policies for these ten states were reviewed using the Global Drug Policy Index and correlated with mortality trends.

**Results:**

Opioid overdose deaths totaled 405,682 nationally, with the crude death rate peaking at 29.8 per 100,000 in 2022. Mortality was highest among males, adults aged 35–44, and Black or African American individuals. West Virginia, Alaska, Washington, Tennessee, and Oregon had the steepest increases, while New Jersey, Utah, New Hampshire, Maryland, and South Dakota showed the most stable or improving trends. Less punitive policy scores (*r* = 0.76, *p* = 0.011) and high proportionality of the criminal justice system (*r* = 0.41, *p* = 0.239) were correlated with rising trends in death rates, while harm reduction (*r* = −0.53, *p* = 0.112) and access to painkillers (*r* = −0.52, *p* = 0.118) showed negative correlations.

**Conclusions:**

Opioid mortality increased nationally during the study period. Among the ten purposively selected states, higher policy scores for the Absence of Extreme Responses (i.e., less punitive) were associated with worsening mortality trends. Whereas higher policy scores for Harm Reduction and Access to Medicines were associated with more favorable mortality trends, although these associations were not statistically significant. This small, extreme-case study demonstrated the feasibility of conducting in-depth analyses of multiple drug policy domains and generated hypotheses for testing in a larger study including all U.S. states.

## Highlights

• Mortality burden was highest among males, adults aged 35–44, and Black or African American individuals.
• West Virginia, Alaska, Washington, Tennessee, and Oregon had the steepest increases; New Jersey, Utah, New Hampshire, Maryland, and South Dakota showed improving trends.
• States with lower punitive drug policy scores experienced a significantly steeper increase in opioid mortality.
• States with higher scores on harm reduction and access to pain medications showed the most favorable declines in opioid mortality.
• A balanced public health and enforcement approach is needed to address the opioid crisis.

## 1 Introduction

The United States (U.S.) has the highest annual number of drug overdose deaths in the world, according to the World Health Organization (World Health Organization, n.d.; Cook, 2022). From 2020 to 2024, more than 76,000 people died from drug overdoses each year, with 76% of these fatalities linked to opioids. The opioid overdose epidemic has worsened over the past two decades, with opioid-related deaths increasing six-fold between 1999 and 2017. In 2017, this crisis was officially declared a national public health emergency by the U.S. Department of Health and Human Services (Hancocks, 2019; The Lancet, 2021).

In the United States, the opioid crisis has unfolded in four distinct waves, each driven by different factors (Ciccarone, 2021). The first wave, beginning in the mid-1990s, was fueled by a significant increase in opioid prescriptions, which were widely promoted as safe and effective for pain management (Centers for Disease Control and Prevention, 2024; Hancocks, 2019; Kline et al., 2024). This shift was influenced by multiple factors, including corporate interests, intense marketing efforts by pharmaceutical companies, and a shift in medical culture that identified pain as the fifth vital sign and emphasized on pain management in clinical care (Cook, 2022; Humphreys et al., 2022; The Lancet, 2021). While seeking to alleviate patients' suffering, doctors frequently prescribed these medications in high doses and for prolonged durations, increasing the risk of opioid use disorders and overdose. Around 2010, a second wave of opioid use disorders began as heroin markets resurged to cater to individuals who had developed dependencies on prescription opioids (Humphreys et al., 2022). The third wave started in 2013, driven by illicit fentanyl and other highly potent synthetic opioids, which are cheaper to produce and widely accessible (Ciccarone, 2019, Ciccarone, 2021; Wilson, 2020). The majority of illicit fentanyl-related deaths in 2019–2020 involved males and surged notably in the Midwest, South, and West regions (O'Donnell, 2021). The “fourth wave” of the U.S. overdose crisis (Ciccarone, 2021; Friedman & Shover, 2023; Jenkins, 2021), beginning around 2020, is characterized by the rapid increase in stimulant co-involvement (e.g. methamphetamine, cocaine) in fentanyl-related overdose deaths. Between 2010 and 2021, the proportion of overdose deaths involving both fentanyl and stimulants rose more than fifty-fold, from 0.6% to over 32% (Friedman & Shover, 2023). By 2021, stimulants were the most common drug class detected alongside synthetic opioids, such as fentanyl, reflecting a shift from single drug to polysubstance overdose risk.

Policy responses to the opioid crisis have been implemented at both federal and state levels, though their effectiveness remains unclear. At the federal level, the U.S. Department of Health and Human Services increased funding and launched comprehensive initiatives focused on substance use prevention, treatment, and recovery services (Benzon et al., n.d.; Food and Drug Administration, 2024). A key component has been expanding access to medication-assisted treatment and naloxone (Monnat, 2022). Naloxone access laws were introduced to increase its availability in non-clinical settings, including provisions for healthcare providers to prescribe it to family members of individuals with opioid use disorder (Lee et al., 2021; Nelson et al., 2022). At the state level, policies have varied widely and include Prescription Drug Monitoring Programs (PDMPs), prescription limit laws, harm reduction services, such as syringe exchange programs, and Good Samaritan laws that provide legal protections for individuals seeking emergency help during overdoses (Judd et al., n.d.; Humphreys et al., 2022; Lee et al., 2021; Sahebi-Fakhrabad et al., 2023; The Lancet, 2021).

Despite these efforts, critical gaps remain in our understanding of how state-level drug policies relate to opioid overdose mortality trends. Policy implementation, however, has been inconsistent across states, and the scope and the impact of these policies vary widely (Sahebi-Fakhrabad et al., 2023; Whitmore et al., 2019). Additionally, less is known about how the policy environment may be associated with trends in synthetic opioid deaths in recent years, particularly during the third and fourth waves of the epidemic when fentanyl and polysubstance use became predominant (Ciccarone, 2021; Friedman & Shover, 2023). Such policy differences may interact with demographic and geographic disparities to produce uneven patterns of opioid-related mortality across the U.S.

This study aimed to address these knowledge gaps by (i) analyzing trends in U.S. opioid overdose mortality from 2018 to 2023, with attention to demographic and geographic disparities; and (ii) examining the association between state-level drug policy environments and opioid overdose mortality trends. We hypothesized that states with more comprehensive harm reduction and pain management policies would demonstrate more favorable mortality trends compared to states with a singular focus on more punitive or restrictive policy approaches.

## 2 Methods

The overall analytical framework consisted of a four-step workflow. First, we estimated state-specific mortality trend slopes for all 51 jurisdictions (50 states and the District of Columbia) from 2018 to 2023 (Table S1). Second, states were ranked by estimated slopes, and we selected the five steepest-increase and five most-favorable-trend states for further analysis. Third, we calculated the Global Drug Policy Index policy domain scores (Absence of Extreme Responses, Proportionality of Criminal Justice Response, Harm Reduction, and Access to Medicines) for these ten states via desk review. Finally, we examined the associations between each policy domain score and each state's mortality trend slope by calculating Pearson correlation coefficients and fitting simple linear regression models.

### 2.1 Study design and data source

We conducted an ecological study using data from the U.S. Centers for Disease Control and Prevention's Wide-ranging Online Data for Epidemiologic Research (CDC WONDER) (Centers for Disease Control and Prevention, n.d.). This publicly available health information system provides comprehensive mortality data at national, state, and local levels and is widely used for epidemiological research, public health planning, and policy evaluation (Centers for Disease Control and Prevention, n.d.).

### 2.2 Definition of opioid overdose death

Drug overdose deaths were identified using *International Classification of Diseases, 10th Revision (ICD-10)* underlying cause-of-death codes X40-X44 (unintentional poisonings), X60-X64 (intentional poisonings), X85 (assault by poisoning), and Y10-Y14 (poisonings of undetermined intent) (Butelman et al., 2023). Opioid overdose deaths were further classified based on multiple cause-of-death codes as follows: Any opioid (T40.0-T40.4, T40.6), heroin (T40.1), prescription opioids (T40.2 for natural and semi-synthetic opioids, and T40.3 for methadone), synthetic opioids other than methadone (T40.4), and other and unspecified narcotics (T40.6).

We reported trends in overall opioid overdose mortality crude rates per 100,000 from populations across 51 jurisdictions from 2018 to 2023. Mortality was stratified by sex (female, male), age groups (15–24 years, 25–34 years, 35–44 years, 45–54 years, 55–64 years, 65+ years), race (American Indian or Alaska Native, Asian, Black or African American, White, more than one race), and Hispanic origin (Hispanic or Latino, not Hispanic or Latino). CDC WONDER data were unavailable (NA) for the following state-ethnicity combinations because counts were suppressed for small sample sizes (*N* < 10) (Table 1): American Indian or Alaska Native in West Virginia; Asian in West Virginia and Alaska; American Indian or Alaska Native in New Hampshire; Asian in New Hampshire and South Dakota; and individuals reporting more than one race in South Dakota. Also, it was unavailable for 65+ years in South Dakota. These suppressed subgroup estimates did not affect analyses of overall state-level trends, as state-level counts were sufficient and no state-level estimates were suppressed.

**Table 1:** Demographic Characteristics of Opioid Overdose Mortality Rate Across Five States with the Worst Trends in the United States.

	West Virginia	Alaska	Washington	Tennessee	Oregon	All States
Overall rate	6078 (67.9)	937 (26.8)	9243 (24.4)	14,255 (41.9)	4291 (20.3)	405,682 (25.0)
**Year**						
2018	701 (46.6)	68 (11.7)	737 (12)	1307 (23.7)	339 (9.8)	46,726 (17.5)
2019	673 (45)	82 (14.1)	825 (13.3)	1538 (27.6)	331 (9.5)	49,766 (18.6)
2020	1127 (75.6)	112 (19.3)	1188 (18.9)	2411 (42.8)	499 (14.1)	68,478 (25.4)
2021	1251 (84)	201 (34.5)	1621 (25.5)	3033 (53.2)	775 (21.9)	80,184 (29.6)
2022	1145 (77)	187 (31.9)	2051 (31.9)	3052 (52.7)	967 (27.2)	81,498 (29.8)
2023	1181 (79.6)	287 (48.9)	2821 (43.7)	2914 (49.9)	1380 (38.8)	79,030 (28.7)

**Gender**						
Female	1902 (42)	334 (20.1)	2689 (14.2)	4581 (26.1)	1232 (11.5)	118,872 (14.4)
Male	4176 (94.4)	603 (32.8)	6554 (34.6)	9674 (58.6)	3059 (29.3)	286,810 (36.0)

**Age groups**						
15–24 years	297 (22.6)	87 (15)	821 (14.6)	1047 (19.6)	361 (11.7)	30,309 (11.7)
25–34 years	1458 (113.6)	248 (35.8)	2096 (29.7)	3604 (62.5)	1058 (29.5)	101,752 (37.1)
35–44 years	1849 (144.8)	251 (41.9)	2198 (34.2)	4044 (77.3)	1058 (30.3)	106,290 (41.4)
45–54 years	1364 (100.3)	167 (33)	1741 (31.2)	3055 (58.3)	772 (24.9)	80,353 (32.9)
55–64 years	882 (59.3)	143 (26.4)	1755 (30.9)	2033 (37.6)	717 (22.7)	66,381 (26.1)
65+	224 (11.2)	41 (10.6)	632 (8.4)	467 (7.3)	325 (6.9)	20,597 (6.1)

**Ethnicity**						
Hispanic or Latino	42 (27.8)	36 (15.6)	921 (20.9)	440 (25.2)	379 (14.8)	49,548 (17.7)
Not Hispanic or Latino	6016 (68.4)	891 (27.3)	8257 (24.7)	13,772 (42.6)	3859 (20.8)	353,342 (26.3)

**Race**						
Black or African American	395 (121)	42 (32.4)	830 (50.8)	2750 (49.6)	209 (45.6)	73,523 (34.5)
White	5646 (67.3)	525 (22.2)	7229 (24.1)	11,354 (41.9)	3754 (20.4)	317,948 (25.4)
American Indian or Alaska Native	NA	261 (51.9)	488 (74.7)	20 (12.6)	132 (36.3)	5102 (25.8)
Asian	NA	NA	184 (4.7)	47 (7)	38 (3.6)	3266 (3.2)
More than one race	27 (21.4)	104 (49.9)	453 (30.4)	76 (14.5)	146 (20.5)	5439 (15.0)

The numbers in each cell represent N (Crude Rate per 100,000); NA: suppressed data bellow ten (*N* < 10).

### 2.3 Selection of states for policy analysis

For the policy analysis, we ranked states by the slope of opioid overdose mortality trends between 2018 and 2023 using linear regression models. We used an extreme case analysis approach (Cochrane, n.d.) and limited the study to ten states in total due to time and feasibility considerations, in which five states with the steepest increases and the five states with the most stable or improving trends were selected for further analysis (Fig. 1a & 1b). This approach allowed us to contrast states with the highest and lowest mortality trends.

**Fig. 1:**
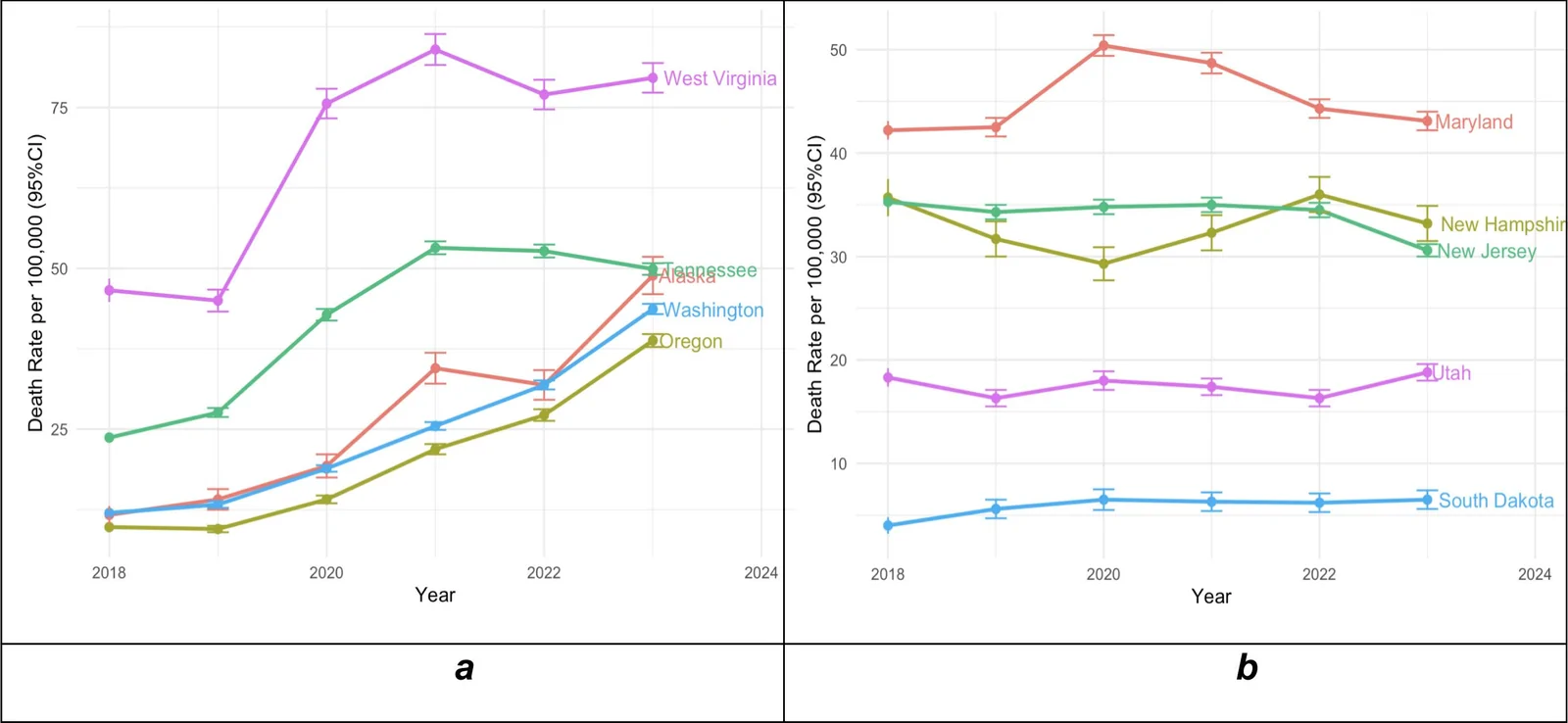
Five states with the worst (sharply increasing) trends [Fig. 1a] and five states with the best (most improving or stable) trends [Fig. 1b].

### 2.4 Drug policy framework

State-level drug policies were assessed using the Global Drug Policy Index (GDPI). The original use of the GDPI model selected 75 policy indicators based on a systematic review of drug policies and expert consultations. The index was created by the Harm Reduction Consortium, led by the International Drug Policy Consortium, including 192 civil society and community organizations. The Harm Reduction Consortium was working in partnership with Swansea University (UK) (Thornton, 2021). These indicators included aspects such as criminalization, harm reduction, pain treatment access, human rights considerations, and social impact. To create more manageable and comparable units, the 75 policy indicators were grouped into 21 thematic clusters, each representing a specific aspect of drug policy, such as law enforcement practices, access to pain treatment, and harm reduction services. These 21 policy clusters were then further aggregated into five core policy areas, which define a country's drug policy approach (Harm Reduction Consortium, 2021).

The index was composed of 75 indicators that span five domains (Wall and Bewley-Taylor, 2021):

• **Absence of Extreme Responses**: This domain covered the use of the death penalty, extrajudicial killing, militarized policing, life sentencing, and non-consensual confinement. States with fewer extreme responses received higher scores.
• **Proportionality of Criminal Justice Response**: This domain assessed how the criminal justice system impacts human rights, including issues like excessive sentencing, unnecessary detention, the treatment of drug offenses, and how these issues disproportionately affect vulnerable groups. Higher scores were given where there were no reports of violence or torture by the police, and no disproportionate treatment based on ethnicity, gender, or socioeconomic status.
• **Harm Reduction**: This domain evaluated how states' policies emphasize harm reduction for people who use drugs, including funding, service availability, and equitable access for various populations. States with sustainable funding for harm reduction, and equitable access to harm reduction interventions received higher scores.
• **Access to Medicines**: This domain examined the government's focus on medicine access in its policies, the availability of regulated drugs such as morphine and opioids, and fairness in access for various groups. Better performance in this domain was achieved when policymaking involved key stakeholders and when there were no disparities in access across geographic, gender, or socioeconomic groups.
• **Development**: This domain evaluated countries with sustainable development strategies aimed at reducing illegal crop production. This included analyzing their alternative development policies, methods of crop eradication, and the perceived benefits of these policies for vulnerable groups, such as women, young people, and indigenous communities. We excluded the entire Development domain which aimed at countries with illegal crop production, not relevant to U.S. states.

For this study, the framework was adapted to the U.S. state level by excluding the Development domain, resulting in 64 indicators grouped into 18 clusters within four domains. Each indicator was scored from 0 to 100, where a score of 100 represents strong alignment with human rights and social justice based on the United Nations recommendations (United Nations, 2019).

A multi-level weighting system was used to calculate scores, including indicator-to-cluster weights, cluster-to-dimension weights, and domain-to-overall score weights. At each stage, the scoring and weighting system was conducted by members from the Scientific Advisory Group, composed of individuals with expertise in the academic study and evaluation of global drug policy. The process employed the Delphi method, a structured approach for synthesizing expert opinion through multiple rounds of questionnaires. After each round, experts reviewed the group's aggregated responses and revised their judgments until a refined consensus was reached. This procedure ensured equity and reliability across different regions. For example, the harm reduction funding indicator asked: *“How adequate are levels of harm reduction funding relative to estimated need?”* Responses are classified into five levels: very low, low, moderate low, moderate and adequate. These categories correspond to scores of 20, 40, 60, 80, 100, respectively. Similarly, indicators that asked about the extent of implementation use five-level response options: not at all, to a small extent, to a moderate extent, to a large extent, and to a very large extent, with each level assigned a corresponding score.

For the drug policy analysis, we conducted a desk review of state policies related to the four domains outlined in the GDPI covering the period from 2018 to 2023. Our desk review included available documents, reports, and secondary data such as academic papers, organizational records, government reports, and project documents related to opioid policies available by the end of 2023. The primary academic database searched were PubMed, Embase and Web of science. Indicators for extreme sentencing were informed by data from the *Death Penalty Information Center* (Death Penalty Information Center, n.d.)*,* a national non-profit organization that compiled data on capital punishment and some state legislative sources (New Jersey Legislature, n.d.; U.S. Department of Justice, 2020). Measures of criminal justice proportionality were drawn from U.S. *police violence databases* (Police Scorecard, n.d.)*.* The Medical Subject Headings (MeSH) and keyword terms used for criminal justice indicators included incarcerat*, imprison*, arrest*, detention, detain*, criminal justice, criminal law, convict*, enforcement, enforc*, decriminaliz*. Harm reduction indicators such as opioid agonist therapy and needle/syringe programs were assessed using data from the *Substance Abuse and Mental Health Services Administration (SAMHSA)* (Substance Abuse and Mental Health Services Administration, n.d.) and the *National Survey of Substance Abuse Treatment Services* (The National Survey of Substance Abuse Treatment Services, 2020)*.* Additional search terms used for harm reduction and equity-related indicators included harm reduction, needle*, syringe*, naloxone access, opioid agonist therapy, social determinants, socio-economic, ethnic*, minorities, minority groups, gender, and race. Finally, indicators related to access to pain medication, particularly disparities by race and ethnicity, were derived from the *National Center for Health Statistics* (Centers for Disease Control and Prevention, 2018) and peer-reviewed publications in *PubMed* (Acevedo et al., 2018; Hong et al., 2023; Zajacova et al., 2023).

### 2.5 Statistical analysis

We extracted crude opioid overdose mortality rates (per 100,000 population) from CDC WONDER for all U.S. states covering the years 2018 to 2023. Data cells with fewer than ten deaths were suppressed in accordance with confidentiality requirements (Quick, 2019). The CDC WONDER data includes variables defining drug subtypes, gender, age groups, race, and Hispanic origin. CDC WONDER data were unavailable (NA) for the following state-ethnicity combinations because counts were suppressed for small sample sizes (*N* < 10) (Table 1): American Indian or Alaska Native in West Virginia; Asian in West Virginia and Alaska; American Indian or Alaska Native in New Hampshire; Asian in New Hampshire and South Dakota; and individuals reporting more than one race in South Dakota. Also, it was unavailable for 65+ years in South Dakota. These suppressed subgroup estimates did not affect analyses of overall state-level trends, as state-level counts were sufficient and no state-level estimates were suppressed.

Next, a linear regression model estimated the trend slope of opioid-related mortality rates for each state. States were then ranked from steepest to flattest based on the slope (b coefficient) (Table S1). We selected the top five states with the steepest slope and the bottom five states with the flattest slope for further trend analysis and policy evaluation.

Drug policy evaluation was conducted through a desk review using the adapted GDPI. Each policy indicator was scored from zero to 100, where higher scores indicate stronger alignment with human rights and social justice standards. Indicators were grouped into 18 thematic clusters across four domains (excluding Development). For each state, domain-level and overall GDPI scores were calculated. The data sources and values for the state-level policy indicators are presented in the Supplementary Excel file, including the source. National-level data were used only for 8 indicators out of the 64 total. These indicators were: What is the country's score on the World Bank Fair Trial Indicator?; To what extent does enforcement of drug policy disproportionately impact low-income groups?; How adequate is harm reduction funding relative to estimated need?; To what extent are there disparities due to sexual orientation or gender identity ?; To what extent are there geographic disparities in access to controlled drugs?; To what extent are there gender-based disparities in pain medication access?; To what extent are there disparities in access to controlled drugs for the treatment of pain due to individuals' ethnicity?; and To what extent are there disparities in access to opioid analgesics for the treatment of pain for people who use drugs?

To examine the association between the policy environment and overdose outcomes, we calculated Pearson correlation coefficients (r) and generated scatter plots with fitted linear regression lines and 95% confidence intervals. The fitted regression lines provide a visual summary of the average annual change in crude mortality rates across the 10 states. Pearson correlation coefficients quantified the strength and direction of the linear association between state-level policy domain scores and annual changes in crude mortality rates, while the 95% confidence intervals conveyed the precision of these estimates. Together, these measures were used to assess the relationship between state-level policy domain scores and annual changes in crude overdose mortality rates across the 10 selected states.

All analyses were conducted using R (v.4.4.0) and RStudio (v.2024.4.1.748).

### 2.6 Ethics

This study used de-identified, publicly available secondary data and did not require institutional review board approval. Mortality data were obtained from CDC WONDER as aggregate state-level counts, with CDC disclosure-control procedures (suppression of cells with fewer than 10 deaths) maintained throughout. Policy measures were derived exclusively from publicly available government documents, legislative records, and published reports. Because state-level comparisons may contribute to stigmatization, we interpreted the results in the context of structural, historical, and policy factors rather than as reflecting the characteristics of state populations.

## 3 Results

### 3.1 Demographic characteristics of opioid overdose mortality rate across states

We analyzed the subpopulation (defined by age, gender, race, and ethnicity) trends in the overall and ten selected states (Table 1, Table 2). Between 2018 and 2023, there were a total of 405,682 opioid-related overdose deaths, with a national crude death rate of 25.0 per 100,000 population in the U.S. The rate increased over time, rising from 17.5 in 2018 to a peak of 29.8 in 2022 before declining slightly to 28.7 in 2023. Some states had a higher burden. For example, West Virginia had the highest opioid overdose rate at 67.9 per 100,000, followed by Tennessee (41.9) and Alaska (26.8). In contrast, the lowest rates were reported in South Dakota (5.9), Utah (17.5) and New Hampshire (33.1).

**Table 2:** Demographic Characteristics of Opioid Overdose Mortality Rate Across Five States with the Best Trends in the United States.

	Maryland	New Hampshire	New Jersey	Utah	South Dakota	All States
Overall rate	13,556 (45.2)	2324 (33.1)	15,254 (34.1)	2651 (17.5)	251 (5.9)	405,682 (25.0)
**Year**						
2018	2082 (42.2)	410 (35.7)	2579 (35.3)	437 (18.3)	28 (4)	46,726 (17.5)
2019	2099 (42.5)	365 (31.7)	2499 (34.3)	397 (16.3)	39 (5.6)	49,766 (18.6)
2020	2496 (50.4)	340 (29.3)	2536 (34.8)	446 (18)	46 (6.5)	68,478 (25.4)
2021	2453 (48.7)	382 (32.3)	2665 (35)	446 (17.4)	45 (6.3)	80,184 (29.6)
2022	2241 (44.3)	429 (36)	2634 (34.5)	426 (16.3)	45 (6.2)	81,498 (29.8)
2023	2185 (43.1)	398 (33.2)	2341 (30.6)	499 (18.8)	48 (6.5)	79,030 (28.7)

**Gender**						
Female	3765 (24.2)	674 (19)	3966 (17.2)	1048 (13.9)	107 (5)	118,872 (14.4)
Male	9791 (68.1)	1650 (47.4)	11,288 (51.9)	1603 (21.1)	144 (6.6)	286,810 (36.0)

**Age groups**						
15–24 years	710 (15.6)	137 (13.4)	794 (11.8)	209 (6.4)	44 (6.1)	30,309 (11.7)
25–34 years	2910 (59.1)	657 (62.4)	3611 (51.4)	629 (21.6)	72 (10.5)	101,752 (37.1)
35–44 years	3007 (61.9)	655 (66.4)	3725 (52.5)	733 (26.9)	59 (8.9)	106,290 (41.4)
45–54 years	2979 (63.2)	486 (45.4)	3519 (48.8)	523 (24.9)	36 (6.3)	80,353 (32.9)
55–64 years	3106 (63)	313 (24.2)	2874 (38.6)	392 (21.4)	28 (4)	66,381 (26.1)
65+	836 (15.8)	69 (7.1)	731 (7.9)	165 (7.1)	NA	20,597 (6.1)

**Ethnicity**						
Hispanic or Latino	563 (19.3)	78 (29.4)	2480 (27.9)	279 (13.5)	10 (Unreliable)	49,548 (17.7)
Not Hispanic or Latino	12,953 (47.9)	2234 (33)	12,767 (35.6)	2371 (18.1)	240 (5.8)	353,342 (26.3)

**Race**						
Black or African American	5576 (59.9)	50 (40.8)	3687 (55.4)	46 (20.7)	11 (Unreliable)	73,523 (34.5)
White	7775 (43.9)	2245 (34.2)	11,311 (35)	2484 (18.1)	168 (4.5)	317,948 (25.4)
American Indian or Alaska Native	31 (17.5)	NA	13 (Unreliable)	52 (23.6)	60 (18.9)	5102 (25.8)
Asian	89 (4.3)	NA	141 (3.1)	14 (Unreliable)	NA	3266 (3.2)
More than one race	74 (10.8)	16 (Unreliable)	92 (11.2)	40 (12.2)	NA	5439 (15.0)

The numbers in each cell represent N (Crude Rate per 100,000); NA: suppressed data below ten (*N* < 10).

Nationally, opioid-related overdose deaths were more common among males than females. The crude death rate was 36.0 for males and 14.4 for females. This gender gap was especially large in West Virginia, with 94.4 deaths per 100,000 males compared to 42.0 in females. South Dakota reported the lowest rates for both genders (6.6 for males and 5.0 for females). Across age groups, people aged 35 to 44 had the highest opioid-related overdose mortality rate (41.4 per 100,000), followed by the 25 to 34 group (37.1) and those aged 45 to 54 (32.9). The lowest rates were seen in people aged 65 and older (6.1). West Virginia reported a striking 144.8 deaths per 100,000 among people aged 35 to 44 compared to 8.9 in South Dakota. Racial and ethnic differences were also evident. Nationally, Black or African American individuals had the highest crude death rate at 34.5 per 100,000, followed by American Indian or Alaska Native individuals (25.8) and White individuals (25.4). Hispanic or Latino individuals had a lower opioid-related overdose death rate (17.7) compared to non-Hispanic or Latino individuals (26.3) across the states. The racial burden was especially high in West Virginia, where the rate among Black or African American individuals was 121.0 compared to 67.3 for White individuals. A similar pattern appeared in Oregon, where Black individuals had a rate of 45.6 per 100,000, while White individuals had a rate of 20.4.

Table S1 presents the crude and age-adjusted opioid overdose mortality rates and annual trends by state and their annual slope rank in all States and District of Columbia from 2018 to 2023.

### 3.2 State-level drug policy performance in ten selected states

State-level drug policy scores, as measured by the GDPI, showed a modest difference between states with the worst and best mortality trends (Table 3). On average, states with the least favorable mortality trends scored 73.28, compared to 71.34 among states with the most favorable trends. Alaska achieved the highest overall score at 77.94. Within the worst-performing groups, West Virginia scored the lowest at 66.48. Domain averages in this group were 98.54 for absence of extreme responses domain, 68.31 for proportionality of criminal justice response, 54.63 for harm reduction, and 69.95 for access to medicines. Among the best-performing states, Maryland had the highest score at 76.86, while South Dakota had the lowest (65.79). Domain-specific averages was 92.37 for absence of extreme responses, 59.52 for proportionality of criminal justice response, 60.75 for harm reduction, and 71.96 for access to medicines.

**Table 3:** Drug policy scores in ten states with worst and best trends in the United States.

Five States with the Worst Trends							
Dimension	Weights⁎	WV	AK	WA	TN	OR	Average
Absence of Extreme Responses	26	92.71	100	100	100	100	98.54
Proportionality of Criminal Justice Response	26	56.83	78.35	75.3	60.06	71.01	68.31
Harm Reduction	24	51.03	61.6	51.49	53.78	55.27	54.63
Access to Medicines	24	63.95	69.93	71.96	71.96	71.96	69.95
Drug Policy Score⁎⁎	100	66.48	77.94	75.21	71.79	75	73.28


Five States with the Best Trends							
Dimension	Weights⁎	MD	NH	NJ	UT	SD	Average
Absence of Extreme Responses	26	91.86	93.05	92.03	93.05	91.86	92.37
Proportionality of Criminal Justice Response	26	72.67	61.22	60.98	58.58	44.16	59.52
Harm Reduction	24	70.03	55.51	64.24	59.15	54.81	60.75
Access to Medicines	24	71.96	71.96	71.96	71.96	71.96	71.96
Drug Policy Score⁎⁎	100	76.86	70.7	72.47	70.89	65.79	71.34

Note: WV: West Virginia, AK: Alaska, WA: Washington, TN: Tennessee, OR: Oregon, MD: Maryland, NH: New Hampshire, NJ: New Jersey, UT: Utah, SD: South Dakota.

⁎ The weighting of data was conducted by the Scientific Advisory Group (SAG), composed of drug policy analysts. The SAG group combined individuals with expertise in the five dimensions of drug policy and applied the Delphi method to reach a consensus.

⁎⁎ Each state is given a total score ranging from 0 to 100. The score represents the weighted average of all relevant policy dimension scores.

Fig. 2 shows the correlations between four policy domains and annual changes in crude opioid overdose death rates across the 10 states with the best and worst mortality trends from 2018 to 2023. The strongest and statistically significant association was observed for absence of extreme responses (*r* = 0.76, *p*-value = 0.011). The proportionality of criminal justice response domain showed a positive correlation too (*r* = 0.41, *p*-value = 0.239) that was not statistically significant. In contrast, harm reduction (*r* = −0.53, *p*-value = 0.112) and access to medicines (*r* = −0.52, *p*-value = 0.118) both showed negative, though non-significant, correlations.

**Fig. 2:**
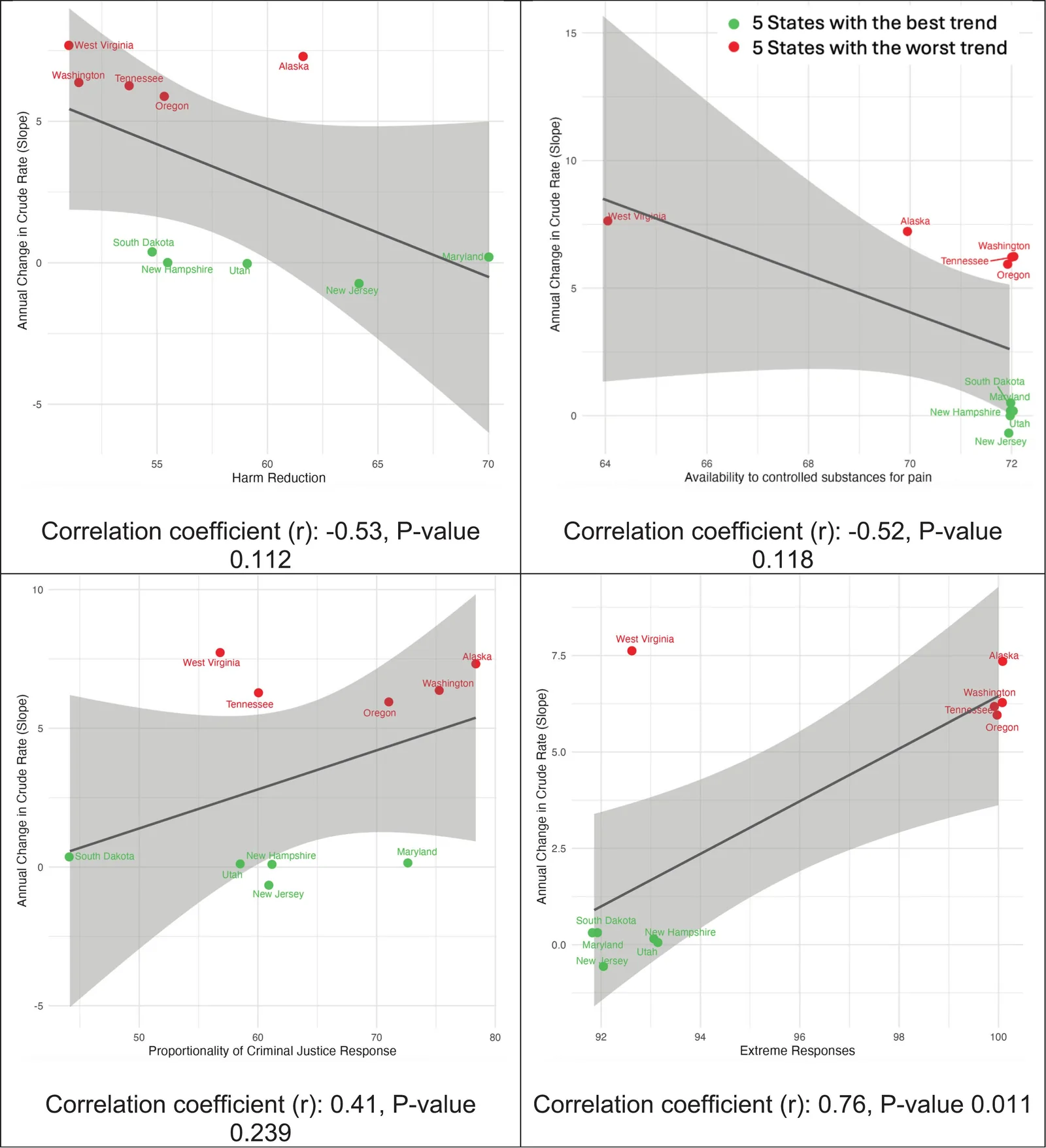
Correlation between policy domains and annual changes in crude death rates.

## 4 Discussion

Our findings revealed ecological correlations suggesting that, among the 10 states examined, those with higher levels of punitive or enforcement-oriented policies (i.e., lower global policy scores on these domains) were associated with greater improvement in overdose mortality trends. We also observed that states with stronger harm reduction or medication access (i.e., higher global policy scores on these domains) appeared to have improving trends in opioid overdose deaths between 2018 and 2023 in the United States. Additionally, opioid overdose mortality continued to rise nationally, with notable differences across states and demographic groups. Opioid overdose mortality was higher among males, adults aged 35 to 44 years, and Black or African American individuals.

Our drug policy analysis revealed a negative correlation between harm reduction policy scores and annual mortality changes (*r* = −0.53, *p* = 0.112), suggesting that states with higher harm reduction scores tended to have more favorable mortality trends, though this association was not statistically significant. However, this non-significant association requires careful interpretation given several important limitations. First, GDPI's harm reduction domain aggregates diverse interventions that vary dramatically in scope and implementation across states. For example, some states maintain extensive needle/syringe program networks (e.g., 50+ programs in California and Massachusetts), while others have severely limited access (e.g., West Virginia had zero legal programs until 2021). Similarly, naloxone access policies range from comprehensive approaches (e.g., standing orders, pharmacy dispensing, third-party prescribing, and layperson administration) to restrictive prescription-only models. Correctional treatment access also varied substantially, from comprehensive approaches (e.g., New Hampshire and Utah offering all FDA-approved medications in state prisons, and Maryland's HB0116 requiring assessment and treatment availability (Maryland General Assembly, 2020)) to more limited programs in other jurisdictions. Additionally, funding stability ranged from multi-year commitments (e.g., South Dakota and New Jersey securing sustained pharmaceutical or governmental funding (South Dakota Department of Social Services, n.d.; State of New Jersey, 2024)) to significant cuts (e.g., Washington losing $160 million in federal substance use disorder funding (Washington State Health Care Authority, n.d.)). Second, our GDPI framework may not capture newer harm reduction tools critical to addressing the fourth wave of the overdose crisis, such as fentanyl test strips, xylazine test strips, and drug checking services, which have emerged as essential interventions but were not systematically assessed in our policy evaluation. Some Absence of Extreme Responses indicators (e.g., death penalty, militarized/military drug enforcement) show little to no state-level variation in the U.S. context. No states currently use the death penalty or military involvement in drug enforcement for drug offenses, meaning that domain's variation across our ten states was driven mainly by differential use of life imprisonment for trafficking offenses (e.g., Utah and New Jersey). These limitations, combined with potential unmeasured confounding, suggest caution in interpreting these ecological correlations as evidence of policy effectiveness.

Our analysis revealed a negative correlation between controlled substance access policy scores and annual mortality changes (*r* = −0.52, *p* = 0.118), suggesting that states with higher access scores tended to have more favorable improvement in mortality trends, though this association was not statistically significant. All 10 selected states had explicit provisions for controlled medicines, yet we found that disparities in access to pain management remained, particularly in West Virginia and Alaska. Contributing factors included geography, socioeconomic status, and race or ethnicity. For example, West Virginia is located in the Appalachian Mountains, where healthcare services are less accessible, which contributes to low primary healthcare access for 30% of the population (Hong et al., 2023). In addition, studies have shown that rural residents often face longer travel distances to reach opioid treatment programs compared to those in urban areas (Acevedo et al., 2018; Amiri et al., 2024). Racial and ethnic disparities also persist, as national studies point out that Black and Hispanic populations have a lower likelihood of receiving any analgesia compared to White populations due to the long-term consequences of structural violence (Rengifo et al., n.d.; Schuler et al., 2020). Notably, these same geographic, socioeconomic, and racial factors that create access disparities are also strong predictors of overdose mortality and were not controlled for in our analysis. Although direct evidence on the availability and accessibility of controlled substances was limited in the remaining states, the policy domain on access to controlled medicines within the GDPI framework captures the broader social determinants of health that influence equitable access to pain medication such as opioids. These findings highlight the complex interplay between policy environments and social determinants of health, though our ecological design precludes determination of whether policy differences or underlying population characteristics drive the observed associations.

For the “absence of extreme response domain” of our policy framework, we observed a counterintuitive and statistically significant association (*r* = 0.76, *p* = 0.011) whereby states with the best improvement in the mortality trends generally had lower policy scores, indicating more punitive policies. Although no states currently use the death penalty or military involvement in drug enforcement, some states still impose life imprisonment for certain drug offenses, particularly for leaders of narcotics trafficking networks. For example, Utah and New Jersey maintain life imprisonment penalties for fentanyl trafficking, yet this pattern likely reflects unmeasured confounders rather than policy effectiveness. This finding requires careful interpretation as it contradicts extensive research (Bratberg et al., 2023) demonstrating that punitive drug policies typically worsen rather than improve public health outcomes. States with more punitive drug laws may systematically differ in ways we could not measure, including socioeconomic factors, healthcare infrastructure, drug supply patterns, or demographic composition-all known predictors of overdose mortality. For example, a cohort of 3.26 million U.S. adults (2008 to 2019) showed that incarcerated individuals had a 39% higher all-cause and over threefold higher overdose mortality risk than non-incarcerated people. The study also showed that counties with higher jail incarceration rates had increased mortality among residents (Khatri et al., 2025).

Between 2018 and 2023, the U.S. faced a sharp rising trend in opioid-related overdose deaths, totaling 405,682 (crude rate at 25.0 per 100,000), with wide variation across states. West Virginia, Alaska, and Washington showed the steepest annual increases (slope range 6.31 to 7.70), while New Jersey, Utah, and New Hampshire (slope range − 0.65 to 0.10) had the most stable or improving trends. Demographically, men had over double the crude death rate of women, consistent with previous studies showing that men are more likely to die from drug overdoses due to a combination of biological, behavioral, and social factors (Butelman et al., 2023; Hanson et al., 2020; Hedegaard & Warner, 2021; Monnat, 2022). Adults aged 35 to 44 had the highest age-specific mortality, likely reflecting both high drug tolerance and accumulated socioeconomic stressors (Spencer et al., n.d.; Monnat, 2022). Racially, Black people experienced the highest overdose death rates due to long-standing structural racism and inequities in access to effective treatment (Cohen et al., n.d.; Han et al., 2022; Monnat, 2022; Schoenthaler & Williams, 2022).

However, these geographic and demographic patterns likely reflect multiple unmeasured factors beyond policy differences. Most notably, the temporal diffusion of fentanyl markets from East to West (2013−2020) may explain our state groupings more parsimoniously than policy differences; Eastern states in our ‘best outcomes’ group experienced earlier fentanyl saturation and are now plateauing, while Western states in our ‘worst outcomes’ group saw explosive fentanyl penetration during our study period (2018–2021). Our study period also coincided with unprecedented COVID-19 disruptions (2020−2021) that differentially affected harm reduction services across states, with some maintaining programs while others experienced closures. Nationally, opioid overdose mortality followed a similar pattern, with the crude rate rising from 17.5 per 100,000 in 2018 to a peak of 29.8 in 2022 before declining to 28.7 in 2023. This rise-and-plateau pattern is consistent with well-documented pandemic-era disruptions to treatment and harm reduction access nationally, and that our state-level policy correlations should be interpreted with this shared, non-policy driver of the 2020–2022 surge in mind. Simultaneously, massive opioid settlement funding from companies such as Purdue and Janssen (2019–2023) provided billions in resources with highly variable spending patterns across states during our outcome period. Furthermore, states differed substantially in baseline healthcare infrastructure and demographic composition. For example, states with robust pre-crisis healthcare systems (e.g., high insurance coverage, strong primary care networks, existing substance use disorders treatment capacity) were better positioned to respond effectively.

The geographic patterns also reflect the evolution toward fourth-wave polysubstance overdoses, where Western states' established methamphetamine markets created complex challenges when fentanyl penetrated these regions (2018–2020). This evolution toward polysubstance use patterns presents challenges for traditional harm reduction interventions designed for earlier waves (e.g., naloxone which is less effective for opioid-stimulant combinations, opioid agonist therapy which does not address stimulant use, and needle/syringe programs which are less relevant as smoking becomes more common) may have reduced effectiveness in addressing fourth-wave polysubstance overdoses. Similarly, GDPI's pain management policy domain becomes increasingly irrelevant when over 90% of opioid deaths by 2020 involved illicitly manufactured fentanyl rather than prescription opioids. Collectively, these factors suggest that observed correlations may reflect the complex interplay of temporal epidemic evolution, healthcare system capacity, funding variations, and pandemic disruptions rather than policy effectiveness per se.

This study has several important limitations that affect the interpretation of our findings. First, our findings should be interpreted as exploratory and hypothesis-generating only. The ecological correlation design and absence of control for key sociodemographic, economic, and structural confounders preclude causal inferences about policy effectiveness. Additionally, the study's design limitations prevent us from making policy recommendations based on these results. This represents a major limitation as these unmeasured confounders may explain the observed associations between policy scores and mortality trends, limiting our ability to infer policy effects. Additionally, our extreme case analysis approach, focusing on only 10 states with the most divergent trends, limits generalizability to other states, and that the small sample size (*n* = 10) limits statistical power and the precision of our estimates. Second, due to missing data for eight state-level indicators of drug policies, we used national-level data instead. This may have introduced inconsistencies and limited the comparability across states. Third, our evaluation of drug policy performance was based on a desk review of governmental and organizational websites. If a state did not publish or update the latest policies online, we may have missed provisional or ongoing changes. Additionally, some policy indicators were not opioid-specific. Fourth, while we examined opioid-related overdose mortality between 2018 and 2023, our review of drug policies was not restricted to this period and may have included more recent policies post 2023. This difference in timing could be a confounding factor, as we aimed to evaluate whether drug policies were associated with opioid overdose deaths between 2018 and 2023. For example, one indicator involved assessing the sustainability of harm reduction funding over the next 3–5 years. We included data as recent as 2025 in some states to better reflect future-oriented planning. Finally, we applied the original GDPI weighting structure, developed through an international Delphi consensus process, rather than deriving U.S.-specific weights. As a result, the relative importance assigned to indicators may not fully reflect their relevance to opioid overdose mortality in the U.S. context.

Despite the limitations, this is to our knowledge, one of the first attempts to adapt the GDPI framework for sub-national (U.S. state-level) comparison of drug policy environments. This exploratory analysis identified ecological correlations between certain drug policy domains and state-level overdose mortality trends among 10 states with extreme outcomes. The counterintuitive pattern we observed for the Absence of Extreme Responses domain (*r* = 0.76), where states with more punitive scores tended to show more favorable mortality trends, runs contrary to much of the existing literature on punitive drug policy, and is therefore a specific, testable hypothesis (most likely reflecting unmeasured confounding such as fentanyl market diffusion timing, as we discuss) that future controlled, all-state studies should investigate directly.

## CRediT authorship contribution statement

**Yun-Ting Wang:** Writing – original draft, Project administration, Methodology, Investigation, Formal analysis, Data curation. **Said Iftekhar Sadaat:** Writing – review & editing, Validation, Methodology, Investigation, Data curation, Conceptualization. **Daniel Ciccarone:** Writing – review & editing, Validation, Supervision, Methodology, Investigation. **Mohammad Karamouzian:** Writing – review & editing, Validation, Methodology, Investigation, Conceptualization. **Ali Mirzazadeh:** Writing – review & editing, Writing – original draft, Validation, Supervision, Methodology, Investigation, Funding acquisition, Formal analysis, Data curation, Conceptualization.

## Ethical approval

This study used publicly available, de-identified data from the CDC WONDER database and secondary policy data sources. As no human subjects were directly involved, this study was not considered human subjects research and did not require institutional review board approval.

## Funding

This work was supported in part by the 10.13039/100000002National Institutes of Health (NIH) under Grant No. 5R25MH129290-04 (the H2R Training Program). The funder had no role in the study design, data collection and analysis, decision to publish, or preparation of the manuscript. The content is solely the responsibility of the authors and does not necessarily represent the official views of the NIH.

## Declaration of competing interest

The authors declare that they have no known competing financial interests or personal relationships that could have appeared to influence the work reported in this paper.

## Data Availability

the supplementary files containted all data we used in the paper

## Appendix A Supplementary data

Supplementary material 1 For the drug policy indicators
