# Supplementary material for: Drug policies and annual opioid overdose mortality in U.S. states with the five worst and five best trends between 2018 and 2023: an ecological study
Source: Addict Behav Rep. 2026 Oct 22;24:100760. doi: 10.1016/j.abrep.2026.100760 (PMC13720621; doi:10.1016/j.abrep.2026.100760)
Supplement: Supplementary file 1 [file mmc2.docx]

**Table S1. Crude and Age-Adjusted Opioid Overdose Mortality Rates and Annual Trends by State, United States and District of Columbia, 2018-2023**

|  | **Opioid Overdose Crude Mortality Rate (per 100,000)** | | | | | | | |  | **Age-Adjusted Opioid Overdose Mortality Rate (per 100,000)** | | | | | | | |
| --- | --- | --- | --- | --- | --- | --- | --- | --- | --- | --- | --- | --- | --- | --- | --- | --- | --- |
| **State + DC** | **2018** | **2019** | **2020** | **2021** | **2022** | **2023** | **Slope (β)** | **Slope (β) Rank** |  | **2018** | **2019** | **2020** | **2021** | **2022** | **2023** | **Slope (β)** | **Slope (β) Rank** |
| Alabama | 9.5 | 10.3 | 15.1 | 23.7 | 26.3 | 28.6 | 4.35 | 40 |  | 10.5 | 11.3 | 16.9 | 26.8 | 29.7 | 32.6 | 5.02 | 40 |
| Alaska | 11.7 | 14.1 | 19.3 | 34.5 | 31.9 | 48.9 | 7.27 | 50 |  | 11.2 | 13.7 | 19.4 | 35.1 | 31.7 | 50.5 | 7.61 | 50 |
| Arizona | 19 | 21.7 | 30.8 | 33.3 | 31.7 | 31.5 | 2.71 | 26 |  | 20.3 | 23.4 | 33.8 | 36.3 | 34.7 | 34.4 | 3.05 | 28 |
| Arkansas | 8.5 | 8 | 11.6 | 15.7 | 15.6 | 12.5 | 1.34 | 11 |  | 9.3 | 8.8 | 12.7 | 17.4 | 17.4 | 13.8 | 1.51 | 13 |
| California | 7.5 | 10.1 | 17.1 | 22.4 | 22.8 | 24.5 | 3.67 | 38 |  | 7.4 | 10.1 | 17.4 | 22.6 | 23.4 | 25 | 3.80 | 36 |
| Colorado | 12.1 | 13.4 | 20.4 | 26.7 | 24.1 | 26.6 | 3.17 | 32 |  | 12 | 13.4 | 20.8 | 27.4 | 24.5 | 27.4 | 3.34 | 31 |
| Connecticut | 31.9 | 37 | 42 | 46 | 44.4 | 39 | 1.76 | 17 |  | 35.1 | 40.4 | 45.7 | 48.5 | 46.7 | 40.5 | 1.39 | 11 |
| Delaware | 44.3 | 48.1 | 49.8 | 53.8 | 57.7 | 53.1 | 2.19 | 24 |  | 49.9 | 54.7 | 55.9 | 60.9 | 63.8 | 59.7 | 2.32 | 24 |
| D. Columbia (DC) | 32.3 | 40.8 | 56.1 | 61.8 | 60.8 | 61.1 | 5.99 | 47 |  | 34 | 42.7 | 57.5 | 61.9 | 61.8 | 62.6 | 5.85 | 46 |
| Florida | 17.9 | 20.9 | 30 | 32.5 | 29.7 | 26.5 | 2.05 | 23 |  | 20.1 | 23.7 | 34.2 | 36.8 | 33.6 | 29.6 | 2.28 | 23 |
| Georgia | 10.2 | 10 | 15.1 | 20.5 | 22.3 | 20.9 | 2.74 | 27 |  | 10.5 | 10.4 | 15.8 | 21.7 | 23.7 | 22.1 | 2.97 | 27 |
| Hawaii | 5.1 | 4.7 | 6.4 | 7.6 | 8.7 | 11.1 | 1.23 | 10 |  | 5.2 | 4.5 | 6.8 | 7.7 | 9 | 12 | 1.38 | 10 |
| Idaho | 8.7 | 9.3 | 11 | 15.6 | 17.3 | 16.7 | 1.96 | 21 |  | 9 | 9.6 | 11.3 | 16.3 | 18.7 | 18.3 | 2.25 | 22 |
| Illinois | 20.8 | 21.4 | 28.6 | 29.3 | 30.7 | 27.8 | 1.82 | 18 |  | 21.7 | 22.3 | 29.8 | 30.1 | 31.6 | 28.5 | 1.78 | 17 |
| Indiana | 20.4 | 23 | 34.3 | 40 | 37.3 | 30.4 | 2.82 | 29 |  | 22.2 | 25.3 | 38.1 | 43.4 | 40.6 | 33.5 | 3.08 | 29 |
| Iowa | 5.6 | 6.3 | 8.7 | 10 | 9.1 | 9.6 | 0.85 | 8 |  | 6.2 | 6.8 | 9.7 | 10.9 | 10.1 | 10.6 | 0.95 | 8 |
| Kansas | 6.7 | 7.8 | 11 | 18.5 | 21.2 | 17.7 | 2.93 | 30 |  | 7.2 | 8.5 | 11.7 | 20 | 23 | 18.8 | 3.14 | 30 |
| Kentucky | 27.2 | 28.5 | 46.2 | 51.6 | 47.8 | 43.7 | 4.17 | 39 |  | 29.8 | 31.3 | 51.2 | 56.9 | 53 | 48.2 | 4.65 | 39 |
| Louisiana | 11.9 | 14.9 | 25.2 | 35.6 | 36.5 | 30.1 | 4.75 | 43 |  | 12.7 | 16 | 27.4 | 38.6 | 39.9 | 33 | 5.27 | 42 |
| Maine | 24.8 | 28.3 | 36.6 | 46.7 | 52.1 | 43 | 4.93 | 45 |  | 29.7 | 33.6 | 43.3 | 53.7 | 61.1 | 49.9 | 5.54 | 44 |
| Maryland | 42.2 | 42.5 | 50.4 | 48.7 | 44.3 | 43.1 | 0.23 | 4 |  | 42.7 | 43.2 | 51.3 | 48.9 | 44.8 | 43 | 0.11 | 4 |
| Massachusetts | 34.4 | 34 | 35.6 | 38.6 | 39.4 | 35.7 | 0.73 | 7 |  | 37.3 | 36.8 | 39 | 41.4 | 42.1 | 38.1 | 0.64 | 7 |
| Michigan | 24.4 | 21.7 | 26.6 | 30.5 | 29 | 27.6 | 1.19 | 9 |  | 26.5 | 23.5 | 29.1 | 33.1 | 31.5 | 29.7 | 1.26 | 9 |
| Minnesota | 7.6 | 9.4 | 14.8 | 21 | 22 | 21 | 3.17 | 33 |  | 8 | 9.9 | 15.8 | 22.6 | 23.6 | 22.6 | 3.45 | 33 |
| Mississippi | 7.2 | 10.2 | 16.9 | 23.2 | 22.6 | 20.6 | 3.16 | 31 |  | 7.7 | 10.9 | 18.6 | 25.7 | 25.3 | 22.9 | 3.61 | 34 |
| Missouri | 22.7 | 21.8 | 27.4 | 31.2 | 30.9 | 28.4 | 1.70 | 16 |  | 24.9 | 23.9 | 30.3 | 34.2 | 33.8 | 31.4 | 1.89 | 18 |
| Montana | 7.3 | 8 | 9.7 | 12.4 | 14 | 13.6 | 1.49 | 13 |  | 7.9 | 9.1 | 10.9 | 14 | 15.7 | 15.1 | 1.68 | 15 |
| Nebraska | 4.1 | 4.4 | 6.5 | 7.2 | 7.6 | 4.9 | 0.41 | 6 |  | 4.2 | 4.6 | 6.6 | 7.7 | 8.2 | 5.4 | 0.51 | 6 |
| Nevada | 15.1 | 14.9 | 21.8 | 23.5 | 24.2 | 32.6 | 3.35 | 35 |  | 14.6 | 14.8 | 22.6 | 24 | 25.1 | 33.6 | 3.64 | 35 |
| New Hampshire | 35.7 | 31.7 | 29.3 | 32.3 | 36 | 33.2 | 0.10 | 3 |  | 41.9 | 37 | 34.1 | 36.2 | 40.7 | 38.3 | -0.14 | 2 |
| New Jersey | 35.3 | 34.3 | 34.8 | 35 | 34.5 | 30.6 | -0.65 | 1 |  | 37.8 | 36.1 | 36.7 | 36.3 | 35.7 | 31.7 | -0.92 | 1 |
| New Mexico | 19.9 | 23.1 | 31.2 | 43.2 | 41.9 | 40.5 | 4.90 | 44 |  | 21.3 | 25.4 | 34.3 | 47.3 | 46.1 | 45.4 | 5.59 | 45 |
| New York | 18.5 | 18.2 | 26.4 | 30.1 | 32.6 | 32.4 | 3.33 | 34 |  | 19.2 | 19 | 27.7 | 31.2 | 33.9 | 33.3 | 3.39 | 32 |
| North Carolina | 21 | 21.2 | 30.2 | 38.5 | 41.1 | 32.5 | 3.59 | 36 |  | 22.8 | 23 | 33.2 | 42.3 | 45.2 | 36 | 4.05 | 38 |
| North Dakota | 5.9 | 7.1 | 11.5 | 12 | 15.5 | 13.8 | 1.86 | 19 |  | 6.6 | 7.5 | 12.1 | 12.9 | 17 | 14.8 | 2.01 | 19 |
| Ohio | 33.9 | 36 | 45.7 | 46.2 | 43.7 | 39.1 | 1.42 | 12 |  | 37.7 | 40 | 51 | 51 | 48.1 | 43 | 1.45 | 12 |
| Oklahoma | 9.8 | 8.5 | 10 | 14.6 | 23.4 | 25.5 | 3.65 | 37 |  | 9.9 | 8.8 | 10.6 | 15.3 | 25 | 27.5 | 4.04 | 37 |
| Oregon | 9.8 | 9.5 | 14.1 | 21.9 | 27.2 | 38.8 | 5.88 | 46 |  | 10.2 | 9.7 | 15.1 | 23 | 28.5 | 40.8 | 6.21 | 47 |
| Pennsylvania | 26.9 | 28.5 | 36.7 | 37.8 | 36.4 | 33 | 1.58 | 14 |  | 30.2 | 32 | 41.2 | 41.7 | 40.3 | 36 | 1.55 | 14 |
| Rhode Island | 30 | 26.9 | 37.1 | 41.5 | 35.8 | 37.3 | 1.93 | 20 |  | 33 | 29.6 | 40.8 | 45.6 | 39 | 41 | 2.09 | 20 |
| South Carolina | 20 | 20.4 | 32.8 | 40 | 41.8 | 38.5 | 4.68 | 41 |  | 21.7 | 22.4 | 36.4 | 44.4 | 46.5 | 42.4 | 5.25 | 41 |
| South Dakota | 4 | 5.6 | 6.5 | 6.3 | 6.2 | 6.5 | 0.40 | 5 |  | 4.4 | 5.7 | 7.5 | 7 | 6.9 | 7.2 | 0.49 | 5 |
| Tennessee | 23.7 | 27.6 | 42.8 | 53.2 | 52.7 | 49.9 | 6.19 | 48 |  | 25.3 | 29.7 | 46.8 | 57.8 | 57.5 | 54.3 | 6.84 | 49 |
| Texas | 6.2 | 6.5 | 9.1 | 11.8 | 13.2 | 13 | 1.62 | 15 |  | 6.2 | 6.5 | 9.2 | 11.9 | 13.5 | 13.3 | 1.69 | 16 |
| Utah | 18.3 | 16.3 | 18 | 17.4 | 16.3 | 18.8 | 0.05 | 2 |  | 18.8 | 16.9 | 18.7 | 17.9 | 17.1 | 19.6 | 0.11 | 3 |
| Vermont | 23.9 | 21.5 | 29.6 | 39.6 | 43 | 41.8 | 4.69 | 42 |  | 29 | 26.4 | 34.9 | 47.6 | 51.8 | 48.6 | 5.34 | 43 |
| Virginia | 17 | 18.1 | 26.9 | 31.4 | 29.3 | 28.9 | 2.79 | 28 |  | 18.1 | 19.2 | 28.9 | 33 | 31.1 | 30.6 | 2.92 | 26 |
| Washington | 12 | 13.3 | 18.9 | 25.5 | 31.9 | 43.7 | 6.31 | 49 |  | 12 | 13.3 | 19.5 | 26 | 32.7 | 44.3 | 6.46 | 48 |
| West Virginia | 46.6 | 45 | 75.6 | 84 | 77 | 79.6 | 7.70 | 51 |  | 53.9 | 52.7 | 88.9 | 98.2 | 89.7 | 90.9 | 8.72 | 51 |
| Wisconsin | 17.7 | 19.4 | 26.1 | 29.5 | 29.2 | 28.9 | 2.54 | 25 |  | 19.4 | 21.1 | 29.3 | 32.9 | 32.7 | 31.8 | 2.87 | 25 |
| Wyoming | 8.6 | 10.1 | 12.7 | 15.1 | 17 | 17.9 | 1.99 | 22 |  | 8.7 | 10.6 | 13.5 | 15.8 | 17.8 | 19.1 | 2.17 | 21 |

Slope (β): Estimated annual change in mortality rate (2018-2023). Rank: Ordered by slope, with 1 = lowest slope and the highest rank = highest slope. The data came from the CDC WONDER Mortality Statistics database. The query included opioid-related underlying cause of death (UCD) and multiple cause of death (MCD) ICD-10 codes, was grouped by residence state and year, and calculated rates per 100,000. For the age-adjusted rate it is using the 2000 U.S. standard population. UCD ICD-10 codes: X40, X41, X42, X43, X44, X60, X61, X62, X63, X64, X85, Y10, Y11, Y12, Y13, and Y14. MCD ICD-10 codes: T40.0 (opium), T40.1 (heroin), T40.2 (other opioids), T40.3 (methadone), T40.4 (other synthetic narcotics), and T40.6 (other and unspecified narcotics).

**Excel Appendix**

Two supplementary Excel files were included with the submission:

- Global Drug Policy Index (GDPI) 2021 Tool
- Global Drug Policy Index (GDPI) for 10 U.S. States - Scoring and Source of Data
